# Establishment of a humanized mouse model of keloid diseases following the migration of patient immune cells to the lesion: Patient-derived keloid xenograft (PDKX) model

**DOI:** 10.1038/s12276-023-01045-6

**Published:** 2023-08-01

**Authors:** A Ram Lee, Seon-Yeong Lee, Jeong Won Choi, In Gyu Um, Hyun Sik Na, Jung Ho Lee, Mi-La Cho

**Affiliations:** 1 Lab of Translational ImmunoMedicine, Catholic Research Institute of Medical Science, College of Medicine, College of Medicine, The Catholic University of Korea, Seoul, South Korea; 2 Department of Biomedicine & Health Sciences, College of Medicine, The Catholic University of Korea, Seoul, South Korea; 3 The Rheumatism Research Center, Catholic Research Institute of Medical Science, College of Medicine, The Catholic University of Korea, Seoul, South Korea; 4 Department of Plastic and Reconstructive Surgery, College of Medicine, The Catholic University of Korea, Seoul, South Korea; 5 Department of Medical Life Sciences, College of Medicine, The Catholic University of Korea, 222, Banpo-daero, Seocho-gu, Seoul, 06591 South Korea

**Keywords:** Experimental models of disease, Lymphocyte activation

## Abstract

Keloid disorder is an abnormal fibroproliferative reaction that can occur on any area of skin, and it can impair the quality of life of affected individuals. To investigate the pathogenesis and develop a treatment strategy, a preclinical animal model of keloid disorder is needed. However, keloid disorder is unique to humans, and the development of an animal model of keloid disorder is highly problematic. We developed the patient-derived keloid xenograft (PDKX), which is a humanized mouse model, and compared it to the traditional mouse xenograft model (transplantation of only keloid lesions). To establish the PDKX model, peripheral mononuclear cells (PBMCs) from ten keloid patients or five healthy control subjects were injected into NOD/SCID/IL-2Rγnull mice, and their keloid lesions were grafted onto the back after the engraftment of immune cells (transplantation of keloid lesions and KP PBMCs or HC PBMCs). Four weeks after surgery, the grafted keloid lesion was subjected to histologic evaluation. Compared to the traditional model, neotissue formed along the margin of the grafted skin, and lymphocyte infiltration and collagen synthesis were significantly elevated in the PDKX model. The neotissue sites resembled the margin areas of keloids in several respects. In detail, the levels of human Th17 cells, IL-17, HIF-1a, and chemokines were significantly elevated in the neotissue of the PDKX model. Furthermore, the weight of the keloid lesion was increased significantly in the PDKX model, which was due to the proinflammatory microenvironment of the keloid lesion. We confirmed that our patient-derived keloid xenograft (PDKX) model mimicked keloid disorder by recapitulating the in vivo microenvironment. This model will contribute to the investigation of cellular mechanisms and therapeutic treatments for keloid disorders.

## Dermatology: A model for studying severe scars

An improved animal model could lead to new treatments for keloids, abnormally heightened scarring responses that can occur after recovery from skin injury. These poorly understood scars occur only occur in humans, and existing animal models are based on immunodeficient mice. This is a serious drawback, given that immune cell invasion appears to be an important contributor to keloid formation. Researchers led by Mi La Cho and Jung Ho Lee at The Catholic University of Korea, Seoul, have devised an improved model that addresses this limitation. Their approach involves infusing mice with human T cells before introducing a graft of patient-derived keloid tissue. These animals subsequently develop scars that resemble human keloids much more closely than those in prior models, offering a powerful tool for studying these enigmatic lesions.

## Introduction

Although keloid lesions are benign, they display aggressive and uncontrolled growth and a high rate of recurrence. These lesions can occur on any area of skin; the most commonly affected sites are the anterior chest, shoulders, back, and earlobe^1^. In most cases, the affected sites are accompanied by pain, disfigurements, and pruritis, which can cause emotional distress and other psychosocial symptoms^2^.

The pathophysiology of keloid disorder is unclear, and effective treatments that do not induce recurrence are not available. The etiology may encompass both genetic and environmental factors. Cytokines such as transforming growth factor (TGF), insulin-like growth factor, and vascular endothelial growth factor are highly expressed in keloid tissue^3^. In addition, proinflammatory cytokines generated by chronic inflammation are associated with pathologic fibroproliferative responses in keloid tissue^4,5^.

To investigate the pathogenesis of keloid disorder and develop a treatment strategy, a preclinical animal model is needed. However, keloid lesions develop only in humans, and the development of animal models is problematic. To overcome this difficulty, the keloid xenograft model, in which keloid lesions are grafted onto the skin or in a subcutaneous pocket of an immune-deficient mouse, was developed^6^. In this model, the grafted keloid lesion remained viable for several weeks to months with evidence of angiogenesis and fibrosis^7,8^; this model has been applied extensively in keloid research^9–11^. However, because keloids lack T and B cells, the microenvironment may not reflect the inflammatory process of keloid disorder.

We developed a new humanized mouse model known as the patient-derived keloid xenograft (PDKX) model and compared it to the mouse xenograft model. To establish the PDKX model, peripheral blood mononuclear cells (PBMCs) from a keloid patient were injected into NOD.Cg-Prkdc^scid^ Il2rg^tm1Wjl^/SzJ (NSG) mice and keloid tissue were grafted onto the back 4 weeks after immune cell transplantation. Four weeks after surgery, the grafted keloid tissue was subjected to histologic analysis.

## Materials and methods

### Keloid lesions and peripheral blood of patients

This study was approved by the Institutional Review Board of Bucheon St. Mary’s Hospital (HC18TESI0013). Keloid lesions (*n* = 10) and PBMCs (*n* = 10) were obtained from 10 keloid patients who underwent surgery for ear keloid lesions. Healthy PBMCs (*n* = 5) were obtained from five healthy controls in this study. Informed consent was obtained from all patients according to the principles of the Declaration of Helsinki.

### Mice

Eight-week-old NSG male mice were purchased from Jackson Laboratories (Bar Harbor, ME). All experimental procedures were approved by the Department of Laboratory Animals, Institutional Animal Care and Use Committee (IACUC) of the School of Medicine, Catholic University of Korea, and conformed with the guidelines of the United States National Institutes of Health (Permit Number: 2021-0027-02).

### Animal experimental modeling

**Fig. 1:**
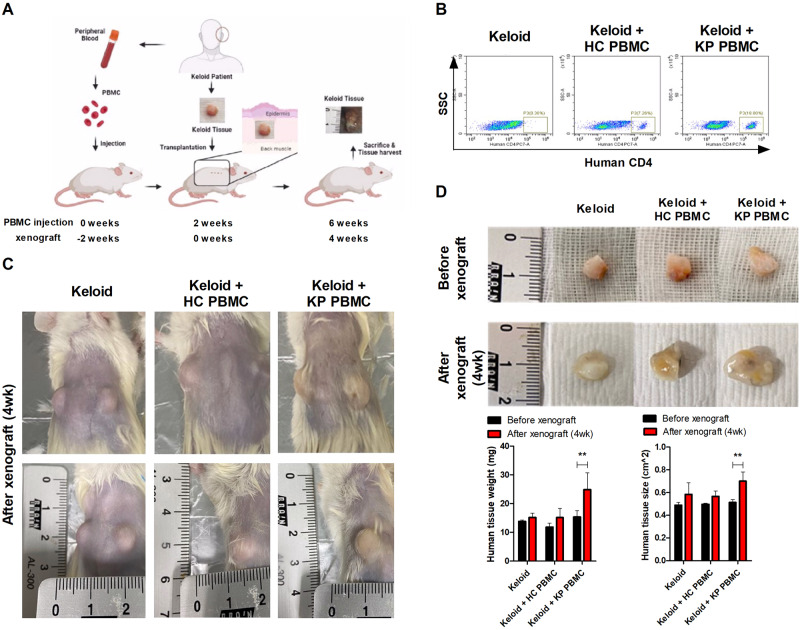
Development of the PDKX model through the transplantation of human keloid PBMCs and tissue. **a** Schematic representation of the humanized mouse model of keloid disorder. PBMCs isolated from the blood of keloid patients (KF, *n* = 10) and healthy controls (HC, *n* = 5) were injected intraperitoneally into NSG mice (3 × 10^6^ cells per mouse). Two weeks later, the keloid lesion was transplanted into the back of the NSG mouse and observed for 4 weeks. **b** Peripheral blood samples of the mice have analyzed for human CD4+ T-cell engraftment by flow cytometry. **c** and **d** Four weeks after xenograft, the transplanted tissues were harvested, and the size and weight were measured. The bar graphs are from three independent experiments; means ± SDs. **p < 0.01.

PBMCs were obtained from the blood of five healthy controls and ten keloid patients and intraperitoneally injected into NSG mice at a dose of 3 × 10^6^/mouse. Two weeks after transplantation, a keloid lesion (0.5 cm^3^, 130–150 mg) was transplanted into the back under the panniculus carnosus muscle. In the control group, keloid lesions were transplanted without preoperative PBMC transplantation. Four weeks after tissue transplantation, the mice were euthanized, and the transplanted human keloid skin lesion was removed for histologic evaluation (Fig. 1a).

### Measurement of keloid lesions

Before transplantation and 4 weeks after skin tissue transplantation, the transplanted grafts were excised and subjected to morphologic analysis, as well as weight and size measurement using an electronic microbalance and ruler.

### Flow cytometry

To confirm the engraftment of human immune cells in mouse blood, flow cytometry was performed. To confirm the engraftment of human CD4 T cells, mononuclear cells in mouse blood were stained with human anti-CD4-PeCy7 (Cat. no. 300511; Biolegend, San Diego, CA). Four weeks after tissue transplantation, IL-17-expressing human CD4^+^ T cells (Th17) were analyzed in the whole blood of mice. Mononuclear cells in mouse blood were stained with human anti-CD4 and IL-17-PE (12-7179-42; eBioscience, San Diego, CA). Before intracellular staining, the cells were stimulated for 4 h with phorbol myristate acetate (25 ng/mL) and ionomycin (250 ng/mL) in the presence of GolgiStop (554715; BD Biosciences, San Jose, CA). Intracellular staining was performed using a BD Cytofix/Cytoperm Plus Fixation/Permeabilization Kit and BD Golgistop Kit (554715; BD Biosciences). Flow cytometry was performed using a cytoFLEX flow cytometer (Beckman Coulter, Brea, CA), and the data was analyzed using FlowJo software (Tree Star, Ashland, OR).

### Histological analysis

Explanted tissue was subjected to histological analyses to evaluate inflammation and collagen density.

The grafted tissues were excised and fixed in 4% neutral buffered formalin for 24 h, embedded in paraffin, and sectioned at a thickness of 5-µm. The tissue slides were deparaffinized and stained with hematoxylin and eosin (H&E). Masson’s trichrome (MT) staining was performed to examine collagen deposition using a Masson’s trichrome staining kit (PolySciences, Inc., Warrington, PA)^12,13^. Inflammation in H&E-stained tissue sections was assigned an ordinal score from 0 to 3 for mononuclear cell infiltration (grade 0: none; grade 1: mild; grade 2: moderate; and grade 3: severe). Collagen in Masson’s trichrome-stained sections was quantified using ImageJ software.

### Immunohistochemistry (IHC)

The tissue sections were incubated at 4 °C with the following primary monoclonal antibodies: anti-HIF-1α (MA1-516; Thermo Fisher Scientific, Waltham, MA), anti-IL-17 (MAB3171-100; R&D Systems, Minneapolis, MN), anti-CD4 (ab133616; Abcam, Cambridge, UK), anti-IL-4 (PA5-25165; Thermo Fisher Scientific), anti-SDF-1 (ab9797; Abcam), anti-CCL2 (ab9669; Abcam), anti-CCL3 (PA5-47000; Thermo Fisher Scientific), anti-CXCL9 (ab9720; Abcam), anti-TGFβ (ab170874; Abcam), and anti-COL1A1 (PA5-50938; Thermo Fisher Scientific). Subsequently, the sections were exposed to horseradish peroxidase-coupled goat secondary antibodies conjugated to dextran (Dako, Glostrup, Denmark). The neotissue of sections was visualized using DAB + chromogen. Three slides were stained per sample of skin tissue; samples were taken at ≥500 µm intervals. The immunostained sections were examined using a photomicroscope (Olympus, Tokyo, Japan). The DAB-positive area was analyzed by color deconvolution with NIH ImageJ software.

### Confocal microscopy

CD4, IL-17, IL-4, pSTAT3 (y705), CXCR3, and procollagen levels were analyzed by confocal microscopy. Paraffin-embedded sections were incubated with 10% normal goat serum for 30 min and stained with anti-CD4 (AF-379-NA; R&D Systems), anti-IL-17 (MAB3171-100; R&D Systems), anti-IL-4 (PA5-25165; Thermo Fisher Scientific), anti-pSTAT3 (y705) (ab76315; Abcam), anti-CXCR3 (SC-133087; Santa Cruz Biotechnology, Dallas, TX), and anti-procollagen (ab64409; Abcam) antibodies. Next, the samples were reacted with anti-goat IgG-FITC (SC-2024; Santa Cruz Biotechnology), anti-mouse-IgG1-Alexa Fluor 555 (A21127; Invitrogen, Waltham, MA), anti-rabbit-IgG-PE (4050-09; Southern Biotech, Birmingham, AL), anti-rabbit-IgG APC (A-10931; Invitrogen), anti-mouse-IgG-Alexa Fluor 647 (1031-31; Southern Biotech), and anti-rat IgG-Alexa Fluor 488 (A-11006; Invitrogen)-conjugated secondary antibodies. Nuclei were stained with DAPI (D3571; Thermo Fisher Scientific). The sections were visualized using a confocal microscope (LSM 700; Carl Zeiss, Oberkochen, Germany); the colocalization area and intensity were analyzed using ZEN 2009 software (Carl Zeiss).

### Statistical analysis

The results are presented as the means ± standard deviations (SD). Data were analyzed using Student’s *t*-test or the Mann‒Whitney *U* test with Prism 5 software (GraphPad, La Jolla, CA); p < 0.05 (two-tailed) was considered indicative of significance.

## Results

### Development of the PDKX model through the transplantation of human keloid PBMCs and tissue

We generated a patient-derived PDKX model as described in the “Materials and methods” section (Fig. 1a). Two weeks after the injection of immune cells from keloid patients or healthy controls into NSG mice, we evaluated the engraftment levels of human CD4 T cells. Human-derived CD4^+^ T cells were identified in the blood of recipient mice. In both PDKX model models that were injected with keloid patient (KP) and healthy control (HC) PBMCs, CD4^+^ T cells were engrafted well. However, the level of engrafted CD4+ T cells was different in the KP PBMC and HC PBMC groups (Fig. 1b). Four weeks after tissue transplantation, the size, and weight of the transplanted tissue were analyzed, and the transplanted tissues were significantly larger in the KP PBMC (9.5 ± 5 mg)-injected group than in the HC PBMC (3.3 ± 2.3 mg)-injected group (Fig. 1c and d).

### Verification of keloid KP PBMCs and the keloid lesion-derived PDKX model by comparison with HC PBMCs and keloid lesions

**Fig. 2:**
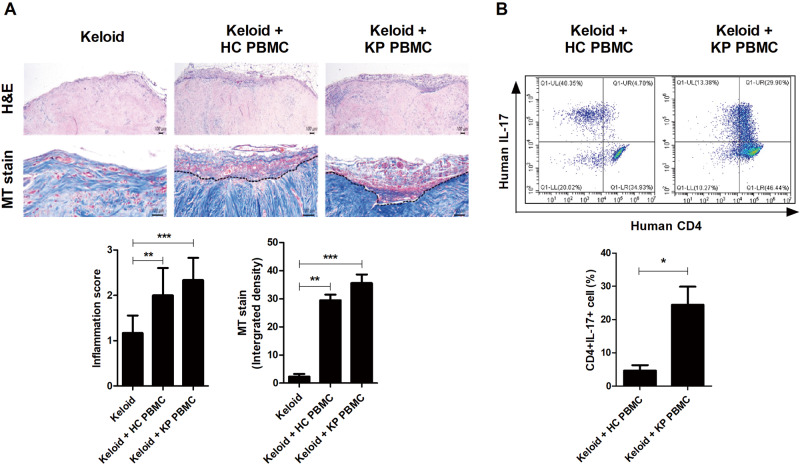
Formation of neotissue along the margin of the grafted skin and lymphocyte infiltration. **a** Representative images of transplanted keloid lesions stained with H&E and MT. Inflammation scores and integrated densities are shown. **b** Mononuclear cells from humanized mouse blood stained with human CD4-PeCy7 and human IL-17-PE. The frequency of human CD4^+^IL-17^+^ T cells is shown. The bar graphs are from three independent experiments; means ± SDs. *p < 0.05; **p < 0.01; ***p < 0.001.

We found neotissue formation along the margin of the grafted skin tissue in the KP PBMC and HC PBMC groups. Interestingly, the neotissue of grafted skin showed the most abundant inflammatory cell infiltration and fibrosis in the KP PBMC group. The transplanted tissues in the KP PBMC PDKX model group had an increased collagen density and collagen bundle sizes (Fig. 2a). The number of CD4^+^IL-17^+^ T cells was significantly increased in KP PBMCs and keloid lesion-transplanted mice (Fig. 2b). We confirmed that neotissue formation along the margin of grafted skin and collagen synthesis was significantly elevated in our PDKX model.

### Evaluation of cytokine expression by cell subtypes in the transplanted keloid tissue of the KP and HC PBMC PDKX models

**Fig. 3:**
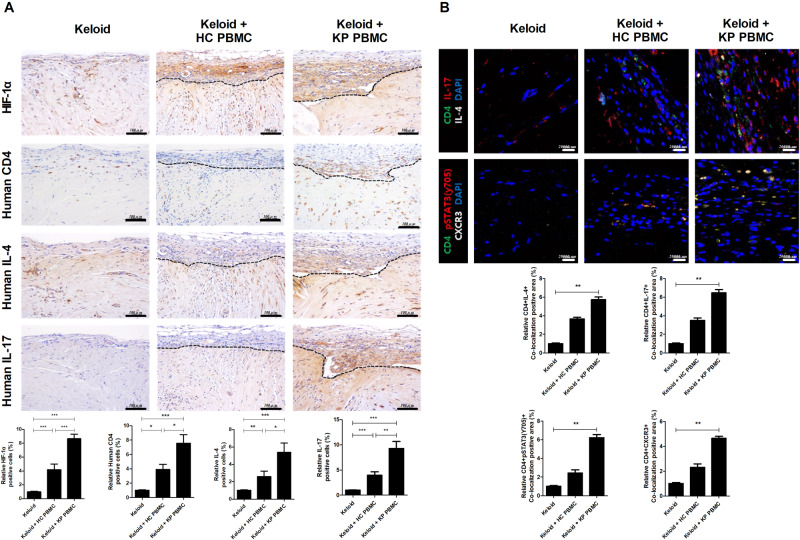
IL-17- and HIF-1α-expressing immune cells were increased in neotissue in the PDKX model. **a** Six weeks after the induction of humanized mice, the transplanted keloid lesions were harvested and analyzed by IHC using antibodies against human HIF-1α, CD4, IL-4, and IL-17. Representative IHC images (upper panels) and graphs (lower panels). **b** Harvested tissues were stained for CD4 (FITC), IL-17 (PE), *p*STAT3y705 (PE), CXCR3 (white), or DAPI (blue). Representative confocal images (upper panels) and graphs (lower panels). Original magnification, ×400; scale bars, 100 μm. Bars represent positive cell numbers per high-power field (HPF); means ± SDs. *p < 0.05; **p < 0.01; ***p < 0.001.

IL-17 expression in T cells is increased in keloid patients and the transitional region of keloid lesions. The IL-17-STAT3-HIF-1α axis is involved in defective homeostasis and increased fibrosis in dermal fibroblasts, implicating IL-17/Th17 cells in keloid disease^14^. IL-4 and IL-17 promote inflammation and fibrosis in systemic sclerosis, pulmonary disease, and liver cirrhosis^15–17^. Therefore, we evaluated cytokine-expressing immune cells in keloid lesions transplanted with KP PBMCs. The numbers of cells expressing human IL-4, IL-17, and HIF1-α were increased in the perigraft area (Fig. 3a). The number of CD4^+^IL-17^+^ T cells was significantly increased in KP PBMCs and keloid lesion-transplanted mice. The numbers of *p*STAT3^705^-positive CD4 T cells and CXCR3-positive CD4 T cells were significantly increased in the perigraft area in the KP PBMC group compared to the HC PBMC and control groups (Fig. 3b). Therefore, Th17 cell infiltration was significantly elevated in our PDKX model.

### T-cell migration was enhanced by CC chemokines in transferred keloid tissue

**Fig. 4:**
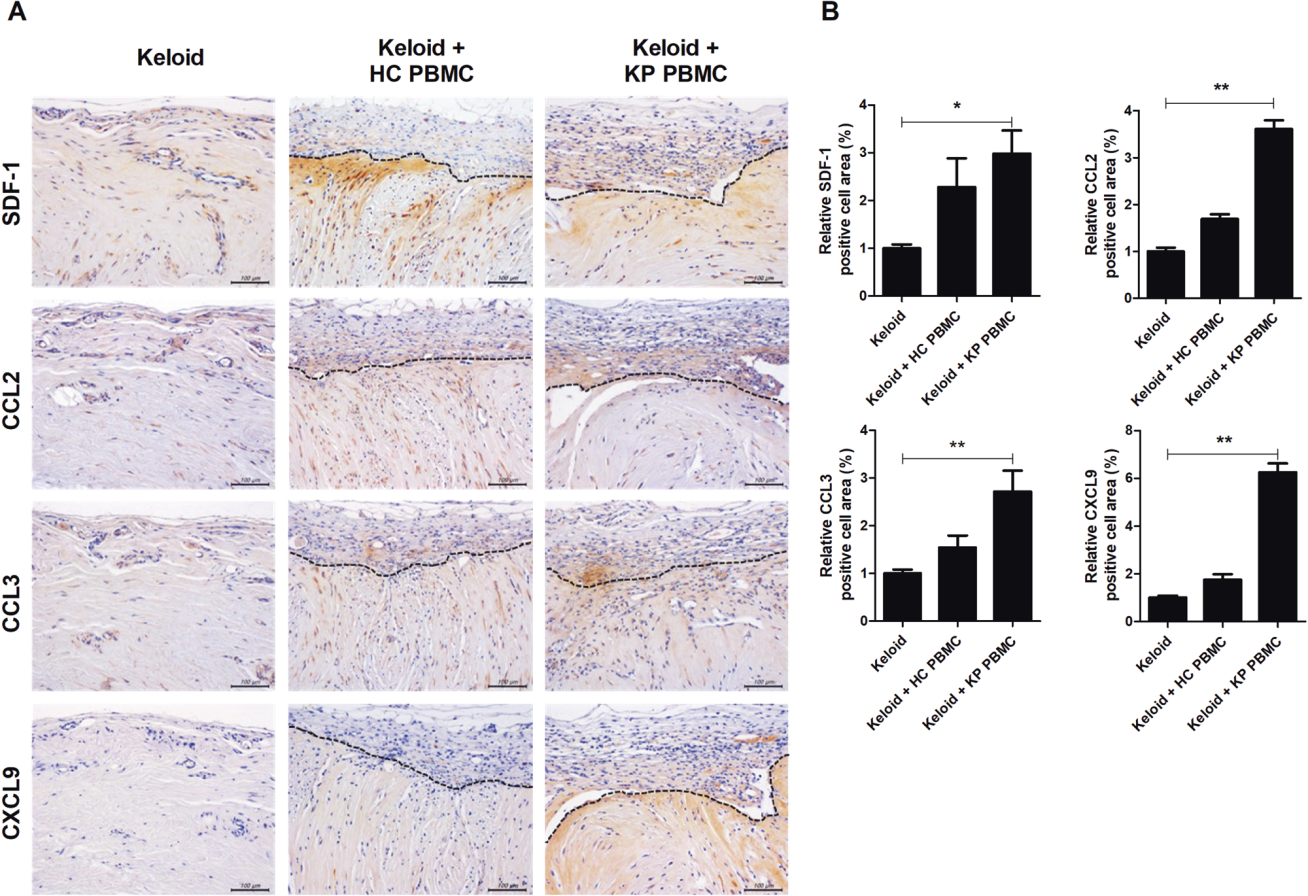
SDF-1 and CC chemokines were elevated in the perigraft and intragraft areas. **a** and **b** Six weeks after the induction of humanized mice, the transplanted tissues were harvested and analyzed by IHC using antibodies against human SDF-1 CCL2, CCL3, and CXCL9. Representative IHC images and graphs. Original magnification, ×400; scale bars, 100 μm. Bars are positive cell numbers per HPF; means ± SDs. *p < 0.05; **p < 0.01; ***p < 0.001.

SDF-1 expression is significantly elevated in the keloid margin area, enhancing the recruitment of Th17 cells^5^. We found that SDF-1, CCL2, CCL3, and CXCL9 expression was significantly increased in the perigraft area in the KP PBMC group (Fig. 4a and b). Chemokines promote inflammation and fibrosis in keloid disorders^18^. Activated fibroblasts and endothelial cells release CCL2, thereby recruiting monocytes and inducing the production of extracellular matrix. The CCL3 level is increased in the plasma of keloid patients^19^. Recruited monocytes also produce other chemokines, such as CXCL9, promoting the infiltration of CXCR3-expressing Th17 cells^20^. Therefore, increased levels of SDF-1 and CC chemokines contribute to the integrity of keloid lesions and promote fibrosis.

### Exacerbation of skin fibrosis in the KP PBMC transplant group by increased procollagen and TGF-β expression

**Fig. 5:**
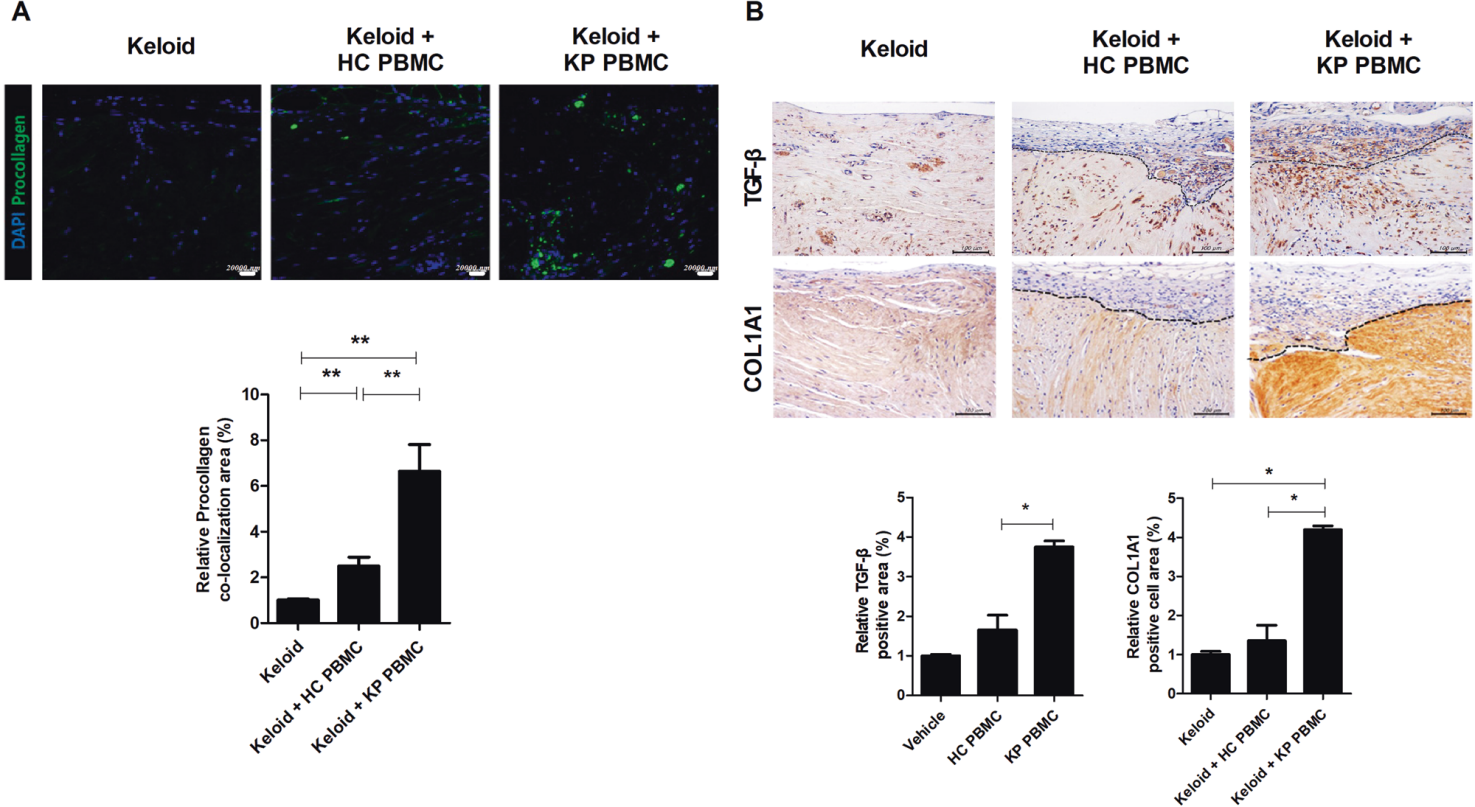
Exacerbation of skin fibrosis in the KP PBMC transplant group due to increased procollagen and TGF-β expression. Six weeks after the induction of humanized mice, the transplanted tissues were harvested and analyzed by confocal microscopy and IHC. **a** The tissues were stained for procollagen (FITC) and DAPI (blue). Representative confocal images (upper panels) and graphs of areas of procollagen and DAPI colocalization (lower panels) are shown. **b** TGF-β and COL1A1 levels were evaluated by IHC. Representative IHC images (upper panels) and graphs (lower panels). Original magnification, ×400; scale bars, 100 μm. Bars are positive cell numbers per HPF; mean ± SD. *p < 0.05; **p < 0.01.

Procollagen is synthesized by fibroblasts and assembled into collagen fibrils^21^. TGF-β and collagen type I alpha 1 (COL1A1) are mediators of fibrosis in keloid patients^22,23^. Procollagen, TGF-β, and COL1A1 expression were significantly increased in the KP PBMC group (Fig. 5a and b). Therefore, our PDKX model accurately reflects chronic inflammation and allows in vivo growth of keloid tissue.

## Discussion

There is no single unifying hypothesis that adequately explains keloid formation; therefore, currently, available treatments include surgical excision and intralesional steroid treatment, but these treatments often induce recurrence^24^. To develop new treatments, greater insight into the molecular pathogenesis of keloid disorder is needed. However, keloid lesions only develop in humans, and so prior studies have used excised keloid lesions or fibroblasts^25^.

In in vivo transplantation-based models, immunodeficient animals (e.g., athymic mice) or an immune-privileged site (e.g., the cheek pouch of a hamster) are used because of the risk of rejection^6,24^. Shetlar^6,7^ described the implantation of keloid lesions into the subcutaneous tissue of athymic mice, and the implanted tissue maintained its integrity for >240 days without rejection. Subsequent keloid research was performed using similar animal models^8,10,26^.

However, these models do not recapitulate the disease microenvironment. The degree of inflammation and characteristics of fibroblasts can be different according to the lesion. For example, lymphocyte infiltration and proinflammatory cytokine (IL-17, IL-1β, IL-6, and tumor necrosis factor-α) expression are increased in the keloid margin area (growing margin) compared to the intralesional or extralesional areas (surrounding normal skin)^5^. Keloid fibroblasts from keloid margin sites show higher collagen I and III expression in vitro than those from intralesional or extralesional sites, and the paracrine effects of inflammatory cells on keloid fibroblasts contribute to disease progression^27,28^. Therefore, to reflect chronic inflammation in keloid disease, the keloid fibroblast–immune cell interaction needs to be modeled.

In this study, we injected T cells into NSG mice 2 weeks prior to the implantation of keloid lesions. Because human T cells were injected into immunocompromised mice, lethal xenogenic graft-versus-host disease (GVHD) could develop within 4–8 weeks^29^. The NSG mouse lacks major histocompatibility complex (MHC) classes I and II, extending the experimental period^30^. In a preliminary experiment, the mice survived for up to 12 weeks without GVHD, and fluorescence-activated cell sorting analysis showed successful engraftment of T cells in 4 weeks. We observed that the level of engrafted CD4^+^ T cells was significantly increased in the KP PBMC group. It has been reported that chimeric human T cells exhibit the phenotype of mature memory cells in a humanized SKID model^31^. In addition, memory T cell infiltration is abnormally present in the scar tissue of keloid patients^32^.

In our PDKX model, neotissue formed along the margin of the grafted skin, and lymphocyte infiltration and collagen synthesis were significantly elevated. The neotissue resembled the keloid margin area of keloids in several respects. First, SDF-1 and IL-17 expression was significantly elevated. Shin et al.^33^ reported high infiltration of SDF-1α + myofibroblasts into keloid margins, which was associated with increased recruitment of CXCR4-expressing immune cells and CXCR4-expressing fibrocytes. Additionally, IL-17 and SDF-1 expression was significantly elevated in the keloid margin area, upregulating SDF-1 and further increasing Th17-cell recruitment, creating a positive feedback loop and excessive fibrosis^5^.

Recently, it was reported that type 3 immunity (IL-17/Th17) is associated with progressive keloid disorder and that IL-17/Th17 induces inflammation and fibrosis in keloid fibroblasts through STAT3/HIF-1α^5,34,35^.

Furthermore, the inflammatory niche-driven IL-17/IL-6 axis is associated with the acquisition by keloid-derived precursor cells of a tumor-like stem cell phenotype^36^. Therefore, IL-17 is important in the pathogenesis of keloids. However, SDF-1 and IL-17 expression was low in the perigraft area in the control group, whereas Th17 cell infiltration and SDF-1 expression were significantly elevated in our PDKX model.

Second, the expression of CC chemokines was significantly elevated in neotissue in the PDKX model. CCL2 stimulates the expression of collagen by fibroblasts and is implicated in renal fibrosis, ischemic cardiomyopathy, atherosclerosis, and pancreatitis^37–39^. CCL2 and CCR2 expression is reportedly enhanced in keloid tissue, increasing fibroblast proliferation^18^. Additionally, CCL3, CCL4, G-CSF, and GM-CSF levels were significantly elevated in the plasma of keloid patients compared to healthy controls, implicating inflammatory cytokines in the formation of keloid lesions^19^. In this study, CCL2 and CCL3 expression was significantly elevated in the perigraft and intragraft areas, contributing to keloid-lesion integrity and augmenting fibrosis. Monocyte chemokines such as CXCL9 promote the infiltration of CXCR3-expressing Th17 cells^20^.

Because keloid disorder is a chronic fibroproliferative disorder, the maintenance or growth of scar tissue in vivo is important. However, Kischer et al. ^8,40^ showed that the size of keloid lesions decreased (slope −0.736) after implantation on the back of an athymic nude mouse, and the volume of keloid lesions decreased by half after 67 days. Waki et al. ^9^ reported that grafts grew rapidly for 4 weeks after implantation and decreased in size thereafter. In this study, the weight of keloid lesions did not change significantly but increased significantly (~1.5-fold) in the keloid + KP PBMC group. This proinflammatory microenvironment is likely responsible for the increase in graft weight. Additionally, the allogeneic immune response was not seen as a concern because the inflammatory response was not found to be significantly higher in the normal PBMC (peripheral blood mononuclear cell) group than in the patient PBMC group.

In conclusion, our PDKX model reflects chronic inflammation in keloid lesions and enables their growth in vivo. This model will contribute to keloid research by recapitulating the in vivo microenvironment.
